# Precious-Metal-Decorated Chromium(IV) Oxide Nanowires as Efficient Catalysts for 2,4-Toluenediamine Synthesis

**DOI:** 10.3390/ijms22115945

**Published:** 2021-05-31

**Authors:** Viktória Hajdu, Alexandra Jakab-Nácsa, Gábor Muránszky, István Kocserha, Béla Fiser, Tibor Ferenczi, Miklós Nagy, Béla Viskolcz, László Vanyorek

**Affiliations:** 1 Institute of Chemistry, University of Miskolc, Miskolc-Egyetemváros, 3515 Miskolc, Hungary; kemviki@uni-miskolc.hu (V.H.); alexandra.nacsa@borsodchem.eu (A.J.-N.); kemmug@uni-miskolc.hu (G.M.); kemfiser@uni-miskolc.hu (B.F.); bela.viskolcz@uni-miskolc.hu (B.V.); 2 Wanhua-Borsodchem, 1 Bolyai tér, 3700 Kazincbarcika, Hungary; 3 Institute of Ceramics and Polymer Engineering, University of Miskolc, Miskolc-Egyetemváros, 3515 Miskolc, Hungary; istvan.kocserha@uni-miskolc.hu; 4 Ferenc Rákóczi II. Transcarpathian Hungarian Institute, UA-90200 Beregszász, Transcarpathia, Ukraine; 5 Institute of Metallurgy, University of Miskolc, Miskolc-Egyetemváros, 3515 Miskolc, Hungary; femft@uni-miskolc.hu

**Keywords:** nanowire, magnetic catalyst, hydrogenation, 2,4-toluenediamine, activation energy

## Abstract

The catalytic hydrogenation of 2,4-dinitrotoluene (DNT) to 2,4-toluenediamine (TDA) is a key step in the production of polyurethanes; therefore, the development of efficient hydrogenation catalysts for industrial use is of paramount importance. In the present study, chromium(IV) oxide nanowires were decorated by palladium and platinum nanoparticles in a one-step, simple, and fast preparation method to yield highly efficient hydrogenation catalysts for immediate use. The nanoparticles were deposited onto the surface of CrO_2_ nanowires by using ultrasonic cavitation and ethanol as a reduction agent. Beneficially, the catalyst became catalytically active right at the end of the preparation and no further treatment was necessary. The activity of the Pd- and Pt-decorated CrO_2_ catalysts were compared in the hydrogenation of 2,4-dinitrotoluene (DNT). Both catalysts have shown high activity in the hydrogenation tests. The DNT conversion exceeded 98% in both cases, whereas the 2,4-toluenediamine (TDA) yields were 99.7 n/n% and 98.8 n/n%, with the Pd/CrO_2_ and Pt/CrO_2_, respectively, at 333 K and 20 bar H_2_ pressure. In the case of the Pt/CrO_2_ catalyst, 304.08 mol of TDA formed with 1 mol Pt after 1 h hydrogenation. Activation energies were also calculated to be approximately 24 kJ∙mol^−1^. Besides their immediate applicability, our catalysts were well dispersible in the reaction medium (methanolic solution of DNT). Moreover, because of their magnetic behavior, the catalysts were easy to handle and remove from the reaction media by using a magnetic field.

## 1. Introduction

Polyurethanes (PUs) are one of the most versatile class of polymers with specific mechanical, physical, biological, and chemical properties giving them great potential for use in different applications, such as flexible and rigid foams, adhesives, paints, coatings, and elastomers. There is an ever-growing demand for toluene diisocyanate (TDI), which is one of the isocyanate components for making PUs; by 2018, global TDI production capacity reached 3.355 million tons per year. The main commercial route for the manufacture of TDI starts with the nitration of toluene using nitric acid to produce dinitrotoluene followed by catalytic hydrogenation to toluene diamine.

2,4-toluenediamine (2,4-TDA) is an important intermediate in the production of toluene diisocyanate (TDI). 2,4-TDA is produced industrially by the catalytic hydrogenation of 2,4-dinitrotoluene (2,4-DNT) in the liquid phase. Numerous catalysts have been developed to produce TDA to achieve the highest possible yield and selectivity in the reaction. The most important catalysts are Raney nickel, carbon-supported palladium or platinum [1,2,3,4,5,6], and silica- [7,8] or alumina-supported [9,10] transition metals.

The rate and selectivity of hydrogenation of aromatic nitro compounds depends on the pressure, temperature, catalyst type, and concentration. As the hydrogen pressure increases, the hydrogenation rate of the nitro compounds also increases [6]. Previous studies on palladium catalysts have concluded that the degree of activity and selectivity depends on the particle size of the palladium. Examination of larger Pd particles has shown that both the specific activity and the selectivity of the 2,4-nitrohydroxiaminotoluene (2,4-HANT) isomers are increased [4]. Industrial applications require a good balance between a catalyst’s performance and its recoverability. Catalyst pellets are easy to recover from the reaction medium; however, the contact surface is smaller between the reagent molecules and the catalyst’s reducing performance. Nanoparticle-based supports offer a high specific surface and good dispersibility; however, as most of the catalysts are powder-based, their separation from the reaction medium is very difficult by conventional methods, such as filtration or centrifugation, after the reaction completed.

This issue can be eliminated by the use of magnetic supports, which is a novel approach in the development of heterogeneous catalytic systems [11,12,13,14,15]. The magnetic support allows easy and efficient separation from the reaction mixture by means of an external magnetic field, thus facilitating the recovery and recyclability [16] of the catalyst. Furthermore, the catalytic activity of the magnetically separable nanoparticles is good because of their large surface area, high stability, and good dispersion in the reaction mixture.

Chromium dioxide (CrO_2_), also known as Magtrieve™, is an extremely interesting metal oxide that can be easily removed from the reaction medium because of its heterogeneous nature and ferromagnetic properties [17]. CrO_2_ films and nanostructures are also important in spintronic applications [18,19,20,21]. Because of its exceptional properties as a magnetic storage medium, it is widely used in audio, video, and computer technology [22]. Furthermore, Magtrieve™ is a magnetically recoverable oxidizing agent also used in organic synthesis [23,24,25], such as for the selective oxidation of alcohols in the form of the corresponding aldehydes, ketones, or carboxylic acids [17,26,27].

Conventional catalyst preparation processes usually consist of several steps including post-treatment to activate the catalysts, which can be avoided by using a sonochemical method [28,29]. By applying sonochemical treatment, an active catalyst can be achieved in only one step [28,29]. The intense ultrasonic irradiation- (sonication) induced sound waves initiate cycles of high and low pressure in the liquid medium. The vapor pressure of the solvent decreases momentarily, which results in the formation of bubbles of a few micrometers in the mixture. These bubbles are pulsating and growing until they reach a higher pressure range in the liquid, where they will collapse as the pressure increases [30]. These are the so-called “hot spots”, where a huge amount of energy is released, causing the liquid phase (i.e., alcohols) to act as a reducing agent in the reaction and initiate the nucleation processes of metal or metal oxide nanoparticles [31,32,33,34]. Here, we report the preparation and investigation of a highly effective and selective magnetic catalyst for DNT hydrogenation. In the present work, palladium and platinum nanoparticles were deposited onto the surface of chromium dioxide in ethanol by using a sonochemical treatment. The activity of the catalysts was tested in 2,4-DNT hydrogenation. Because of the magnetic properties of the catalytic systems, these can be easily and efficiently separated from the reaction mixture by magnetic separation at the end of the hydrogenation process.

## 2. Results and Discussion

### 2.1. Preparation and Characterization of the Pd- and Pt-Decorated Chromium(IV) Oxide Nanowires

**Figure 1:**
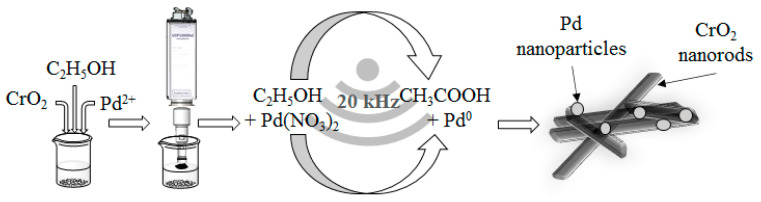
Preparation steps of the Pd/CrO_2_ magnetic catalyst by using a sonochemical treatment.

The preparation of the palladium-decorated magnetic nanowires is highlighted in Figure 1. The detailed procedure for both Pd and Pt is found in the experimental section.

**Figure 2:**
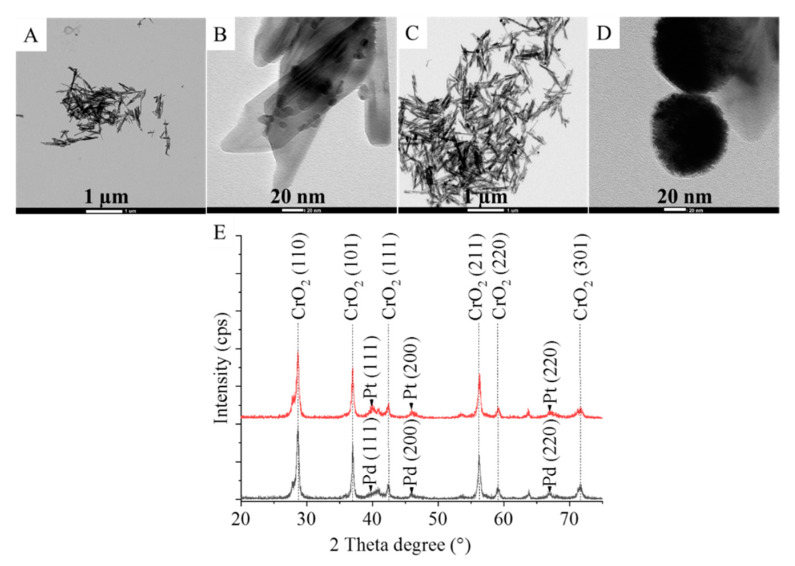
TEM images of (**A**,**B**) the Pd/CrO_2_ and (**C**,**D**) the Pt/CrO_2_ catalysts, and (**E**) the corresponding XRD patterns.

The specific surface area is a key factor related to catalyst performance. Therefore, the specific surface area of the catalysts was determined based on the Brauner–Emmett–Teller (BET) method. There was no significant difference between the Pd/CrO_2_ and Pt/CrO_2_ as their corresponding values were found to be 31.54 m^2^ g^−1^ and 30.08 m^2^ g^−1^, respectively. High-resolution transmission electron microscopy (HRTEM) was also applied in the characterization of the catalysts. The fibrous structure of the chromium dioxide nanowires can be seen on the TEM images, and it was found that their diameter varied between 6 and 60 nm, while their length was between 60 and 870 nm (Figure 2A–D). In the Pd/CrO_2_ system, the palladium nanoparticles were also located on the surface of the CrO_2_ nanowires, and the corresponding particle size was quite small, 6–20 nm (Figure 2B). In contrast, in the case of the platinum-decorated sample, the particles formed larger (70–80 nm) aggregates (Figure 2D).

On closer inspection, it can be observed that the 70–80 nm spherical Pt aggregates build up from several very small (only several nanometers) particles (Figure S1 in the Supplementary Materials). The smaller platinum particles have a significantly larger specific surface with more catalytically active Pt atoms on the surface than in the case of palladium aggregates. These surface atoms are accessible to the reactant molecules (DNT and H_2_), which results in a more efficient transformation, as is described later. Deposition of the two precious metals was different, since the reduction of palladium(II)-ions from the corresponding nitrate salt by ethanol proceeded easily. However, the reduction of Pt(IV)-ions from their stable complex (H_2_PtCl_6_) failed with methanol. Thus, the deposition of platinum particles was realized using another stronger reducing agent, namely, hydrazine solution. The different reduction methods may have resulted in different nucleation and crystallization rates, which could influence the particle size. Consequently, the different reduction methods may explain the aggregate formation of the platinum nanoparticles.

The presence of the elemental palladium was confirmed by XRD measurements. On the diffractogram of the Pd/CrO_2_ catalyst, reflections at 40.2° (111), 46.1° (200), and 66.9° (220) 2θ degrees (JCPDS card number 046–1043) could be seen, which can be associated with the presence of elemental palladium (Figure 2E). The platinum catalyst was also identified and characterized, and reflection peaks were found at 39.8° (111), 45.9° (200), and 67.0° (220) (two theta degrees) (JCPDS card 04-0802), which indicates the presence of elemental platinum (Figure 2E). Thus, the reduction of the Pd(II) and Pt(IV) ions was successful. Additional peaks were also visible on the diffractograms at 28.7°, 36.9°, 42.4°, 56.2°, 59.1°, and 71.7° (two theta degrees), which were identified as the reflections of the (110), (101), (111), (211), (220), and (301) crystalline phases of the catalyst support, magnetic chromium dioxide (JCPDS card number 84-1819).

### 2.2. Comparison of the Catalytic Activity of the Synthesized Pd and Pt Decorated Chromium(IV) Oxide Nanowires

The catalytic activity of the magnetic Pd- and Pt-containing catalysts was tested in DNT hydrogenation at four different reaction temperatures, and the actual DNT concentration was continuously monitored. The precious metal-free CrO_2_ support was also tested at 333 K and 20 bar pressure. In the case of the palladium- and platinum-free CrO_2_, a 53.9 n/n% DNT conversion and a 20.1 n/n% TDA yield were reached after 240 min. The low DNT conversion and TDA yield indicate that Pd or Pt is necessary to achieve a catalytic system of high-performance.

**Figure 3:**
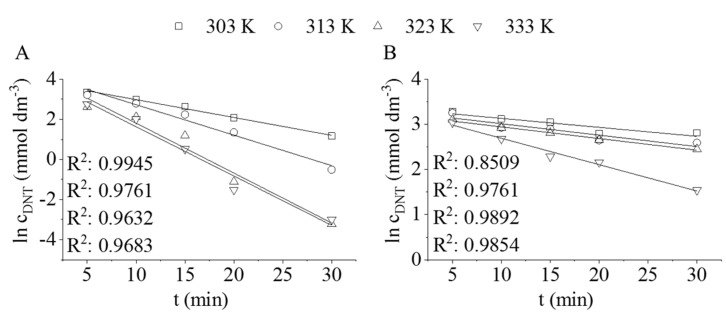
ln(c_DNT_) vs. reaction time diagrams of DNT hydrogenation by applying (**A**) the Pt/CrO_2_ or (**B**) the Pd/CrO_2_ catalyst.

By applying the Pd/CrO_2_ or Pt/CrO_2_ catalyst, the hydrogenation reaction under investigation happens according to a pseudo-first-order kinetics, which was verified by linear regression on the initial experimental points of the ln(c_DNT_) vs. reaction time plot (Figure 3).

**Figure 4:**
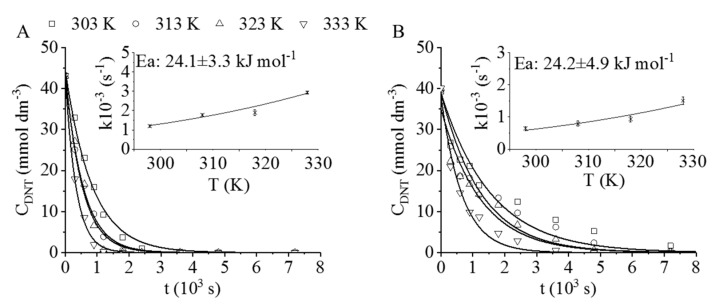
Dinitrotoluene (DNT) conversion (mmol dm^−3^) vs. reaction time measured at four temperatures (303–333 K) and the corresponding Arrhenius plots in the case of (**A**) the Pt/CrO_2_ and (**B**) the Pd/CrO_2_ catalysts.

High DNT conversions were achieved with both catalysts. The platinum-containing sample reacted faster, which can be seen by comparing the steep run of the conversion curves (Figure 4A,B). The system reached full conversion with the Pt/CrO_2_ catalyst after 40 min at 323 K and 20 bar hydrogen pressure. The Pd/CrO_2_ sample was slightly less active than its counterpart, but, after 80 min of hydrogenation at 333 K and 20 bar, it was also able to reach full DNT conversion (Figure 4B).

A non-linear regression method was applied to calculate the reaction rate constants (*k*) (see Equation (3) in the experimental section and Table 1) [35]. In the case of the Pt/CrO_2_, the *k* values were higher (e.g., 2.9 × 10^−3^ s^−1^ at 333 K) compared to the Pd-containing catalyst (e.g., 1.5 × 10^−3^ s^−1^ at 333 K) despite the lower quantity of the precious metal (1.55 × 10^−4^ mol Pt in 1 g catalyst and 4.25 × 10^−4^ mol Pd in 1 g catalyst, respectively).

The activation energies (Ea) were also calculated based on the Arrhenius plot by applying the rate constant (k) vs. temperature (T) diagrams (Figure 4). The fitting was carried out using the original exponential Arrhenius expression (Equation (5)) to avoid the biased error propagation of the parameters (Figure S2A,B). There was no significant difference between the Ea values of the reactions carried out with the tested catalysts (Table 1). However, these activation energies were lower compared to other experimental results in the literature (32.3 ± 7.5 and 38.6 ± 3.3 kJ/mol) [36,37].

The quantity of the adsorbed precious metals was significantly different in the case of the two catalysts. Thus, the catalytic activity of the two magnetic catalysts was compared by using the corresponding turnover numbers (TON) (Equation (4) in the experimental section), which were calculated after 1 h of hydrogenation. In both cases, the TON increased by increasing the temperature, and thus, the highest values were achieved at 333 K. In the case of the Pt/CrO_2_ catalyst, 304.08 mol of TDA formed within 60 min at 333 K and 20 bar pressure (Table 2). The palladium-containing catalyst was less active under the same reaction conditions because only 60.14 mol of TDA formed considering 1 mol of Pd.

**Figure 5:**
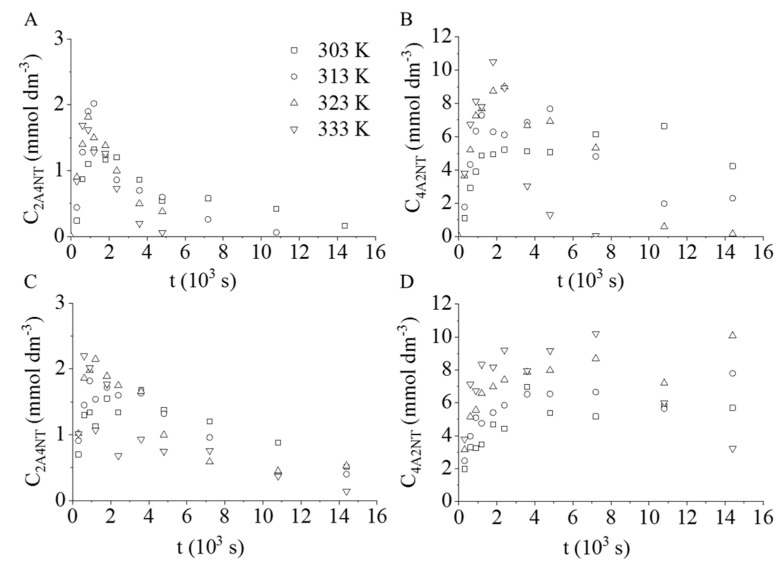
Concentration vs. hydrogenation time of 2-amino-4-nitrotoluene (2A4NT) and 4-amino-2-nitrotoluene (4A2NT) measured in the presence of (**A**,**B**) Pt/CrO_2_ and (**C**,**D**) Pd/CrO_2_, respectively.

The formation of semi-hydrogenated intermediates, 4-amino-2-nitrotoluene (4A2NT) and 2-amino-4-nitrotoluene (2A4NT), were confirmed during the hydrogenation (Figure 5). In the case of the Pt/CrO_2_, the total amount of the intermediates was converted to TDA at higher temperatures (323 K and 333 K). However, the semi-hydrogenated compounds have been detected even after four hours of catalytic hydrogenation with Pd/CrO_2_.

### 2.3. Possible Mechanism for the Hydrogenation

**Figure 6:**
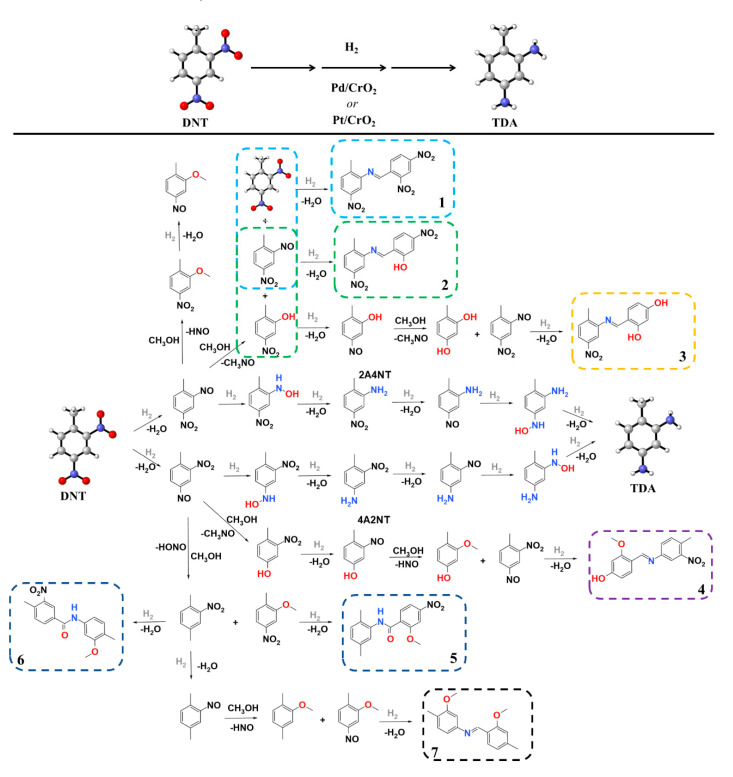
Proposed mechanism of 2,4-dinitrotoluene hydrogenation to produce 2,4-toluenediamine (TDA) with Pd/CrO_2_ or Pt/CrO_2_ catalysts. The reaction pathways depict the formation of specific side-products (dashed frames) detected or assumed during the measurements.

The formation of TDA (Figure 6, middle section) took place through six consecutive hydrogenation steps. In the first step, nitroso-nitrotoluene is formed, which is followed by a hydroxylamine-nitrotoluene formation. This leads to the formation of one of the abovementioned semi-hydrogenated compounds; depending on which nitro group is hydrogenated first, 2A4NT or 4A2NT will be formed. After the other nitro group is hydrogenated, the corresponding nitroso and hydroxylamine compounds will be produced. In the last step, both pathways will lead to the main product, TDA. These results are completely in line with the findings of Giovanni et al. [1], who also detected the presence of nitroso and hydroxylamine compounds during the hydrogenation of DNT over a Pd(C) catalyst. In their case, the two abovementioned compounds further reacted to give 2,2′-dinitro-4,4′-azoxytoluene (DNAT). Our reaction mixture was subjected to Gas Chromatography-Mass Spectrometry (GC–MS) investigation to detect and identify any intermediate present. The possible structures were identified from the MS spectra using the NIST 08, NIST Mass Spectral Library. Much to our surprise, no DNAT or diazo compounds from the reaction of the corresponding amines and nitroxyl-compounds were detected, maybe because of their low stability under GC–MS conditions. Instead, several side-products were identified (Figure 6, see **2**–**4**) in trace amounts in addition to the two semi-hydrogenated intermediates (4A2NT and 2A4NT) during the reaction. The corresponding GC–MS spectra are presented in the Supplementary Materials as Figures S3–S5. In addition to the detected side-products, we assumed the formation of several other ones (**1** and **5**–**7** in Figure 6). The reaction pathways were highlighted to explain the formation of these species. Nota condensation reaction, (*E*)-1-(2,4-dinitrophenyl)-*N*-(2-methyl-5-nitrophenyl)methanimine may form from 2,4-DNT by the loss of water (Figure 6, see **1**). Another condensed molecule, 2-[(*E*)-[(2-methyl-5-nitrophenyl)imino]methyl]-5-nitrophenol was identified, which could be formed through side-reactions by water loss (Figure 6, see **2**). Reaction between the 4-methylbenzene-1,3-diol and the 2-nitroso-4-nitrotoluene led to the formation of 4-[(*E*)-[(2-methyl-5-nitrophenyl)imino]methyl]benzene-1,3-diol (Figure 6, see **3**).

The 2-nitro-4-nitrosotoluene can react with 2-methoxy-4-methylphenol and reach 3-methoxy-4-[(*E*)-[(4-methyl-3-nitrophenyl)imino]methyl]phenol (Figure 6, see **4**). The formation of two stereoisomers, *N*-(2,5-dimethylphenyl)-2-methoxy-4-nitrobenzamide and *N*-(3-methoxy-4-methylphenyl)-4-methyl-3-nitrobenzamide, could occur by the reaction of 1,4-dimethyl-2-nitrobenzene and 2-methoxy-1-methyl-4-nitrobenzene (Figure 6, see **5** and **6**). Moreover, the (*E*)-1-(2-methoxy-4-methylphenyl)-*N*-(3-methoxy-4-methylphenyl)methanimine side-product could form through multiple steps (Figure 6, see **7**).

As it is evident from Figure 6, compounds **1**–**4** and **7** contain imine bonds that can be formed via the reaction of the aromatic amines and the methyl group of the other toluene derivative. This reaction may seem very unlikely at first glance; however, Brandt et al. recently reported the direct alkylation of aliphatic amines via the activation of the sp^3^ C–H bond of pure hydrocarbons, e.g., toluene using an amide-based nickel pincer catalyst [38]. The C–H bonds are very stable by nature, however, alkylation of nitrobenzene with toluene moieties has been reported using a heterogeneous cobalt catalyst in the presence of peroxide and hydrogen. [39] These findings indicate that, by altering the reaction conditions, our catalysts may be also used for the preparation of imines and/or amines by the direct reaction of C–H bonds and amines.

### 2.4. Turnover Number and Reusability

**Figure 7:**
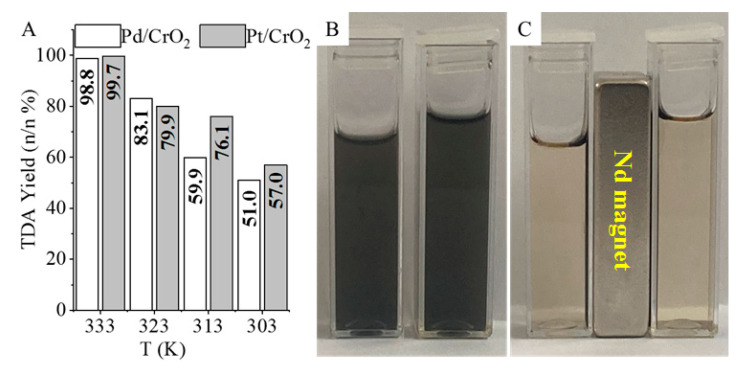
(**A**) TDA yields of the magnetic Pt/CrO_2_ and Pd/CrO_2_ catalysts at different temperatures; (**B**) demonstration of the stable dispersions of the catalysts; and (**C**) their efficient magnetic separation by using a neodymium magnet.

TDA yields were similar in the case of both catalysts, 98.8 n/n% (Pt/CrO_2_) and 99.7 n/n% (Pd/CrO_2_) at 333 K and 20 bar hydrogen pressure despite the fact that the quantities of the precious metals in the catalysts were different (Pt: 3.02 wt%, Pd: 4.52 wt%). At lower temperatures (303 K and 313 K), higher TDA yields (57.0 n/n% and 76.1 n/n%) were achieved by using the Pt/CrO_2_ catalyst, despite its lower metal content. The catalysts were well dispersible in methanolic solution (Figure 7B). Furthermore, because of the magnetic properties of the CrO_2_ support, the separation of the catalysts from the reaction media was easy, efficient, and fast by using a magnetic field (Figure 7C). The Pd/CrO_2_ and Pt/CrO_2_ particles formed stable dispersions with the reaction media in a methanolic solution of DNT and TDA (Figure 7B). The stable catalyst dispersions could be separated using an external magnetic field since the magnetic catalyst particles were collectable by a neodymium magnet from the methanolic phase; therefore, the liquid became clear (Figure 7C).

**Figure 8:**
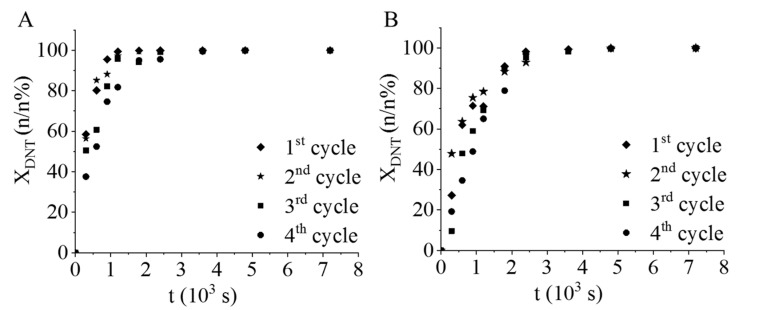
Reuse tests of the catalysts. DNT conversion vs. time by applying (**A**) the Pt/CrO_2_ and (**B**) the Pd/CrO_2_ samples.

Four reusability tests of the catalysts were also carried out without regeneration. Before each reuse test, the catalysts were washed with methanol, and dried at 105 °C overnight; in this sense, the methanolic rinsing was a sufficient post-treatment of the catalysts before the next use. The DNT conversion did not decrease after four cycles, and the maximum conversions were above 99 n/n % in the case of the Pt/CrO_2_ catalyst (Figure 8A).

However, after each cycle, a slight decrease in the slope was seen on the conversion curves, which indicate a small decrease in the reaction rate. A similar tendency was experienced in the case of the Pd/CrO_2_ catalyst, and the DNT conversion was also above 99 n/n% after 60 min. In summary, the catalysts remained active after four cycles without regeneration.

## 3. Materials and Methods

### 3.1. Materials

Palladium(II) nitrate dihydrate (Pd(NO_3_)_2_∙2H_2_O, Merck Ltd., Darmstadt, Germany) and dihydrogen hexachloroplatinate(IV) hydrate (H_2_PtCl_6_∙H_2_O, Alfa Aesar Ltd., Ward Hill, MA, USA) were used as precursors during the catalyst preparation. Patosolv (96–98% ethanol, 2–4% *i*-propanol, Molar Chem. Ltd., Budapest, Hungary) and water were used as solvents; hydrazine hydrate was applied as a reducing agent, while Magtrieve^TM^ chromium(IV) oxide (CrO_2_, Sigma Aldrich Ltd., St. Louis, MO, USA) was used as magnetic catalyst support.

### 3.2. The Preparation of Pd/CrO_2_ and Pt/CrO_2_ Catalysts

Palladium nitrate dihydrate (0.25 g) was dissolved in 50 mL of patosolv and 2.00 g Magtrieve^TM^ was added to the solution (Figure 1). The CrO_2_ was dispersed by using a Hielscher UIP1000hDT Ultrasound Homogenizer for 2 min (115 W, 19.43 kHz). Pd(IV) ions were reduced to elemental Pd nanoparticles. After the reduction, the prepared magnetic Pd/CrO_2_ catalyst was separated from the liquid phase by using a neodymium magnet, and it was washed by patosolv. The magnetic sample was dried at 105 °C overnight.

During the Pt/CrO_2_ catalyst preparation, 0.21 g of dihydrogen hexachloroplatinate(IV) dihydrate was dissolved in 50 mL distilled water, and 2.00 g Magtriev^TM^ was dispersed in the solution by using the homogenizer. Hydrazine hydrate (1 mL) was applied as a reducing agent. The palladium and platinum nanoparticles were deposited onto the chromium(IV) oxide nanowires by adsorption, and the real Pd an Pt contents were checked by Inductively coupled plasma - optical emission spectrometry (ICP-OES) elemental analysis. The precious metal contents of the Pd/CrO_2_ and Pt/CrO_2_ catalyst were 4.52 wt% (4.25 × 10^−4^ mol Pd in 1 g catalyst) and 3.02 wt% (1.55 × 10^−4^ mol Pt in 1 g catalyst), respectively.

### 3.3. Characterization Technics

The morphology and particle size of the palladium- and platinum-decorated CrO_2_ nanowires were examined by high-resolution transmission electron microscopy (HRTEM, FEI Technai G2 electron microscope, 200 kV). Aqueous suspensions of the samples were dosed onto the surface of copper grids (Ted Pella Inc., only carbon, 300 mesh). The particle size of the nanoparticles was measured on the HRTEM images based on the original scale bar by using the ImageJ software. X-ray diffraction (XRD) measurements were applied to identify the metal phases in the catalytic systems by using a Rigaku Miniflex II diffractometer with a Cu Kα radiation source (30 kV, 15 mA). Specific surface area measurements were also carried out by using a nitrogen adsorption–desorption method at 77 K with a Micromeritics ASAP 2020 sorptometer based on the Brauner–Emmett–Teller (BET) method. The quantities of platinum and palladium in the catalysts were measured by using a Varian 720-ES inductively coupled plasma-optical emission spectrometer (ICP-OES). The analysis was carried out by using the Merck Certipur ICP multi-element IV standard. The samples were solved in aqua regia (mixture of nitric acid and hydrochloric acid in a molar ratio of 1:3). The quantitative analysis of the samples was carried out by an Agilent 7890A gas chromatograph coupled with an Agilent 5975C Mass Selective detector. During the measurements, a Restek Rxi-1MS column was used (30 m × 0.25 mm × 0.25 mm). Three analytical standards, 2,4-toluenediamine, 2,4-dinitrotoluene, and 2-methyl-5-nitroaniline (Sigma Aldrich Ltd., Steinheim, Germany) were used in the analysis of the samples. The presence and structure of the intermediates were deduced from the data contained in the NIST 08, NIST Mass Spectral Library.

### 3.4. Catalytic Tests

The prepared chromium(IV)-oxide-supported catalysts were compared in the catalytic hydrogenation of 2,4-dinitrotoluene (DNT). The Büchi Uster Picoclave reactor with a 200 mL stainless steel vessel and heating jacket was used for the tests. Hydrogen pressure was regulated and kept at 20 bar, and the reaction was carried out at 303, 313, 323, and 333 K. Samples were collected after 5, 10, 15, 20, 30, 40, 60, 120, 180, and 240 min. The initial concentration of 2,4-dinitrotoluene was 40 mmol L^−1^ in methanol. Only 0.1 g of catalyst was added to 150 mL of DNT solution. In order to check the catalyst stability, reusability tests were also carried out four times without regeneration.

The concentrations of the main product (TDA), the detected by-products and intermediates were measured by using an Agilent 7890A gas chromatograph coupled with an Agilent 5975C Mass Selective detector. The TDA formation was followed by applying a set of analytical standards (2,4-dinitrotoluene, 2,4-diaminotolune, 2-methyl-5-nitroaniline, 2-methyl-3-nitroaniline, 4-methyl-3-nitroaniline, and 4-methyl-2-nitroaniline, Sigma Aldrich Ltd., Steinheim, Germany). The efficiency of the catalysts was defined by calculating the conversion (*X*%) of DNT based on the following equation (Equation (1)):

(1) $$X\;\%=\frac{consumed\;\;n_{DNT}}{initial\;n_{DNT}}\times 100\;$$

The TDA yield (*Y*%) in case of the catalysts was also calculated (Equation (2)):

(2) $$Y\;\%=\frac{n{\;}_{formed\;TDA}}{n_{\;theoritical\;TDA\;}}\times 100$$

By assuming that the hydrogenation process is a first-order reaction, based on the initial and measured DNT concentrations (*c*_0_ and *c_k_*, mol/dm^3^), the reaction rate constant (*k*) was calculated at different temperatures by applying non-linear regression (Figure 4) as follows (Equation (3)):

(3) $$c_{k}=c_{0}\times \exp\left(-k\times t\right)$$

Turnover numbers (*TON*) were also calculated after 60 min of hydrogenation to compare the activities of the catalysts based on the follow equation (Equation (4)):

(4) $$TON=\frac{n_{TDA}}{n_{catalyst}}$$

where *n_TDA_* is the number of moles of the formed TDA and *n_catalyst_* is the amount of the applied precious metal.

The activation energies (*Ea*) were calculated based on the Arrhenius equation by non-linear regression using (Equation (5)):

(5) $$k=A\times \exp\left[-\left(\frac{Ea}{R\times T}\right)\right]$$

## 4. Conclusions

A fast, efficient, and easy catalyst preparation method was developed to produce magnetic chromium(IV) dioxide nanowires that supported palladium and platinum catalysts. The catalysts showed high catalytic activity in the hydrogenation of 2,4-dinitrotoluene. TDA yields were 99.7 n/n % (Pt/CrO_2_) and 98.8 n/n% (Pd/CrO_2_) at 333 K and 20 bar hydrogen pressure. By GC–MS investigations, trace amounts of side-products were also detected, some of which turned out to be imine derivatives. The imine bonds can be formed via the reaction of the aromatic amines and the methyl group of the other toluene derivative. Based on our experimental results, a possible mechanism for the formation of TDA and the detected side-products was proposed. These findings indicate that, by altering the reaction conditions, our catalysts may be used for the preparation of imines and/or amines by the direct reaction of C–H bonds and amines. Turnover numbers (TONs) were calculated after one hour of hydrogenation, and, in the case of the Pt/CrO_2_ catalyst 304.8 mol of TDA was formed, whereas in the case of the Pd/CrO_2_ catalyst, only 60.14 mol TDA was formed, considering 1 mol of precious metal at 333 K and 20 bar.

In summary, excellent magnetic Pd/CrO_2_ and Pt/CrO_2_ catalysts were achieved and successfully tested in a commercially important hydrogenation reaction. For commercial applications, one of the biggest advantages of these catalysts is their easy and fast preparation, which could reduce catalyst cost. They were well dispersible in the reaction media (methanolic solution of DNT), resulting in a remarkable catalytic activity. In addition, the CrO_2_ magnetic support eliminates the inherent separation issue of nano-formulated catalyst particles. The prepared catalysts can be removed from the medium quickly and completely by using an external magnetic field, which may significantly reduce catalyst loss and, as a result, the production cost. The catalysts can be reused at least four times without regeneration, and GC–MS analysis of the reaction mixture suggests that, by altering the reaction conditions, carbon–nitrogen bonds can also be formed, making these nanoparticles multifunctional catalysts.

## Figures and Tables

**Table 1:** 2,4-Dinitrotoluene hydrogenation reaction rate constants (*k*) calculated at four different temperatures and the corresponding activation energy (E_a_) values achieved by applying the Pt/CrO_2_ and Pd/CrO_2_ catalysts.

	k (s^−1^)				Ea
	303 K	313 K	323 K	333 K	(kJ/mol)
**Pt/CrO_2_**	1.2 × 10^−3^ ± 5.5 × 10^−5^	1.8 × 10^−3^ ± 5.6 × 10^−5^	1.9 × 10^−3^ ± 1.4 × 10^−4^	2.9 × 10^−3^ ± 8.6 × 10^−5^	24.1 ± 3.3
**Pd/CrO_2_**	6.4 × 10^−4^ ± 7.1 × 10^−5^	7.9 × 10^−4^ ± 8.1 × 10^−5^	9.3 × 10^−4^ ± 9.4 × 10^−5^	1.5 × 10^−3^ ± 1.2 × 10^−4^	24.2 ± 4.9

**Table 2:** Turnover numbers (TON) (Equation (4)) after 1 h hydrogenation by using of the Pt/CrO_2_ and Pd/CrO_2_ magnetic catalysts at 303, 313, 323, and 333 K.

	TON [mol_TDA_/mol_Pd/Pt_]			
	303 K	313 K	323 K	333 K
**Pt/CrO_2_**	18.90	85.37	87.89	304.08
**Pd/CrO_2_**	12.71	17.27	43.69	60.14

## Data Availability

Data is available upon request from the corresponding authors.

## Supplementary Materials

The following are available online at https://www.mdpi.com/article/10.3390/ijms22115945/s1.

## References

[1] Neri G., Musolino M.G., Milone C., Visco A.M., Di Mario A. (1995). Mechanism of 2,4-dinitrotoluene hydrogenation over Pd/C. J. Mol. Catal. A Chem..

[2] Neri G., Musolino M.G., Rotondo E., Galvagno S. (1996). Catalytic hydrogenation of 2,4-dinitrotoluene over a Pd/C catalyst: Identification of 2-(hydroxyamino)-4-nitrotoluene (2HA4NT) as reaction intermediate. J. Mol. Catal. A Chem..

[3] Musolino M.G., Neri G., Milone C., Minicò S., Galvagno S. (1998). Liquid chromatographic separation of intermediates of the catalytic hydrogenation of 2,4-dinitrotoluene. J. Chromatogr. A.

[4] Neri G., Musolino M.G., Milone C., Pietropaolo D., Galvagno S. (2001). Particle size effect in the catalytic hydrogenation of 2,4-dinitrotoluene over Pd/C catalysts. Appl. Catal. A Gen..

[5] Neri G., Rizzo G., Milone C., Galvagno S., Musolino M.G., Capannelli G. (2003). Microstructural characterization of doped-Pd/C catalysts for the selective hydrogenation of 2,4-dinitrotoluene to arylhydroxylamines. Appl. Catal. A Gen..

[6] Saboktakin M.R., Tabatabaie R.M., Maharramov A., Ramazanov M.A. (2011). Hydrogenation of 2,4-Dinitrotoluene to 2,4-Diaminotoluene over Platinum Nanoparticles in a High-Pressure Slurry Reactor. Synth. Commun..

[7] Pinna F., Selva M., Signoretto M., Strukul G., Boccuzzi F., Benedetti A., Canton P., Fagherazzi G. (1994). Pd-Fe/SiO_2_ Catalysts in the Hydrogenation of 2,4-Dinitrotoluene. J. Catal..

[8] Benedetti A., Fagherazzi G., Pinna F., Rampazzo G., Selva M., Strukul G. (1991). The influence of a second metal component (Cu, Sn, Fe) on Pd/SiO_2_ activity in the hydrogenation of 2,4-dinitrotoluene. Catal. Lett..

[9] Molga E.J., Westerterp K.R. (1992). Kinetics of the hydrogenation of 2,4-dinitrotoluene over a palladium on alumina catalyst. Chem. Eng. Sci..

[10] Rajashekharam M.V., Nikalje D.D., Jaganathan R., Chaudhari R.V. (1997). Hydrogenation of 2,4-Dinitrotoluene Using a Pd/Al_2_O_3_ Catalyst in a Slurry Reactor: A Molecular Level Approach to Kinetic Modeling and Nonisothermal Effects. Ind. Eng. Chem. Res..

[11] Tsang S.C., Caps V.R., Paraskevas I., Chadwick D., Thompsett D. (2004). Magnetically Separable, Carbon-Supported Nanocatalysts for the Manufacture of Fine Chemicals. Angew. Chem..

[12] Rossi L.M., Costa N.J.S., Silva F.P., Wojcieszak R. (2014). Magnetic nanomaterials in catalysis: Advanced catalysts for magnetic separation and beyond. Green Chem..

[13] Kainz Q.M., Linhardt R., Grass R.N., Vilé G., Pérez-Ramírez J., Stark W.J., Reiser O. (2014). Palladium Nanoparticles Supported on Magnetic Carbon-Coated Cobalt Nanobeads: Highly Active and Recyclable Catalysts for Alkene Hydrogenation. Adv. Funct. Mater..

[14] Stadler L., Homafar M., Hartl A., Najafishirtari S., Colombo M., Zboril R., Martin P., Gawande M.B., Zhi J., Reiser O. (2018). Recyclable Magnetic Microporous Organic Polymer (MOP) Encapsulated with Palladium Nanoparticles and Co/C Nanobeads for Hydrogenation Reactions. ACS Sustain. Chem. Eng..

[15] Purohit G., Rawat D.S., Reiser O. (2019). Palladium Nanocatalysts Encapsulated on Porous Silica @ Magnetic Carbon-Coated Cobalt Nanoparticles for Sustainable Hydrogenation of Nitroarenes, Alkenes and Alkynes. ChemCatChem.

[16] Ye L., Liu X., Lu Y. (2021). A highly controllable, effective, and recyclable magnetic-nanoparticle-supported palladium catalyst for the Suzuki–Miyaura cross-coupling reaction. J. Catal..

[17] Bogdal D., Lukasiewicz M., Pielichowski J., Miciak A., Bednarz S. (2003). Microwave-assisted oxidation of alcohols using Magtrieve™. Tetrahedron.

[18] Ding Y., Yuan C., Wang Z., Liu S., Shi J., Xiong R., Yin D., Lu Z. (2014). Improving thermostability of CrO_2_ thin films by doping with Sn. Appl. Phys. Lett..

[19] West K.G., Osofsky M., Mazin I.I., Dao N.N.H., Wolf S.A., Lu J. (2015). Magnetic properties and spin polarization of Ru doped half metallic CrO_2_. Appl. Phys. Lett..

[20] Solovyev I.V., Kashin I.V., Mazurenko V.V. (2015). Mechanisms and origins of half-metallic ferromagnetism in CrO_2_. Phys. Rev. B.

[21] Duarte A.C., Franco N., Viana A.S., Polushkin N.I., Silvestre A.J., Conde O. (2016). Argon assisted chemical vapor deposition of CrO_2_: An efficient process leading to high quality epitaxial films. J. Alloy. Compd..

[22] Anger G., Halstenberg J., Hochgeschwender K., Scherhag C., Korallus U., Knopf H., Schmidt P., Ohlinger M. (2005). Chromium Compounds. Ullmann’s Encyclopedia of Industrial Chemistry.

[23] Ko K.-Y., Kim J.-Y. (1999). Generation of Diphenyldiazomethane by Oxidation of Benzophenone Hydrazone with Magtrieve TM. Bull. Korean Chem. Soc..

[24] Wan H., Peng Y. (2008). Clean synthesis of azo compounds using Magtrieve™ in the ionic liquid [bmim][Br]. Mon. für Chemie Chem. Mon..

[25] Liu Y.-H. (2008). MagtrieveTM (CrO_2_): A Versatile Oxidant in Organic Synthesis. Synlett.

[26] Lee R.A., Donald D.S. (1997). Magtrieve™ an efficient, magnetically retrievable and recyclable oxidant. Tetrahedron Lett..

[27] Few C.S., Williams K.R., Wagener K.B. (2014). Magtrieve™: A convenient catalyst for the oxidation of alcohols. Tetrahedron Lett..

[28] Prekob Á., Muránszky G., Kocserha I., Fiser B., Kristály F., Halasi G., Kónya Z., Viskolcz B., Vanyorek L. (2019). Sonochemical Deposition of Palladium Nanoparticles Onto the Surface of N-Doped Carbon Nanotubes: A Simplified One-Step Catalyst Production Method. Catal. Lett..

[29] Hajdu V., Prekob Á., Muránszky G., Kocserha I., Kónya Z., Fiser B., Viskolcz B., Vanyorek L. (2020). Catalytic activity of maghemite supported palladium catalyst in nitrobenzene hydrogenation. React. Kinet. Mech. Catal..

[30] Suslick K.S. (2000). Sonochemistry. Kirk-Othmer Encyclopedia of Chemical Technology.

[31] Feng Q.X., Jie Z.J. (2003). Synthesis of Palladium Nanoparticles by A Sonochemical Method. Chin. J. Inorg. Chem..

[32] Qiu X.-F., Zhu J.-J., Chen H.-Y. (2003). Controllable synthesis of nanocrystalline gold assembled whiskery structures via sonochemical route. J. Cryst. Growth.

[33] Ying Y., Qi-Yun Z., Xing-Guo L. (2003). Reduction Process of Transition Metal Ions by Zinc Powder to Prepare Transition Metal Nanopowder. Acta Phys. Chim. Sin..

[34] Wu S.-H., Chen D.-H. (2003). Synthesis and characterization of nickel nanoparticles by hydrazine reduction in ethylene glycol. J. Colloid Interface Sci..

[35] Wang C., Yang F., Yang W., Ren L., Zhang Y., Jia X., Zhang L., Li Y. (2015). PdO nanoparticles enhancing the catalytic activity of Pd/carbon nanotubes for 4-nitrophenol reduction. RSC Adv..

[36] Neri G., Musolino M.G., Milone C., Galvagno S. (2002). Kinetic Modeling of 2,4-Dinitrotoluene Hydrogenation over Pd/C. Ind. Eng. Chem. Res..

[37] Malyala R.V., Chaudhari R.V. (1999). Hydrogenation of 2,4-Dinitrotoluene Using a Supported Ni Catalyst: Reaction Kinetics and Semibatch Slurry Reactor Modeling. Ind. Eng. Chem. Res..

[38] Brandt A., RanguMagar A.B., Szwedo P., Wayland H.A., Parnell C.M., Munshi P., Ghosh A. (2021). Highly economical and direct amination of sp3 carbon using low-cost nickel pincer catalyst. RSC Adv..

[39] Pang S., Yuan H.-K., Wu Y., Shi F. (2017). In Co@N-graphene/C Catalyzed Oxidative Amination of Toluene Derivatives. J. Mol. Catal..

