# Supplementary material for: Precious-Metal-Decorated Chromium(IV) Oxide Nanowires as Efficient Catalysts for 2,4-Toluenediamine Synthesis
Source: Int J Mol Sci. 2021 May 31;22(11):5945. doi: 10.3390/ijms22115945 (PMC8198398; doi:10.3390/ijms22115945)
Supplement: Supplementary file 1 [file ijms-22-05945-s001.zip › ijms-1223084-supplementary.pdf]

SUPPLEMENTARY INFORMATION

Precious Metals Decorated Chromium(IV) oxide Nanowires as Efficient Catalysts for 2,4-toluenediamine Synthesis

Viktória Hajdu[1], Alexandra Jakab-Nácsa[1,2], Gábor Muránszky[1], István Kocserha[3], Béla Fiser[1,4], Tibor Ferenczi[5], Miklós Nagy[1]*, Béla Viskolcz[1], László Vanyorek[1]*

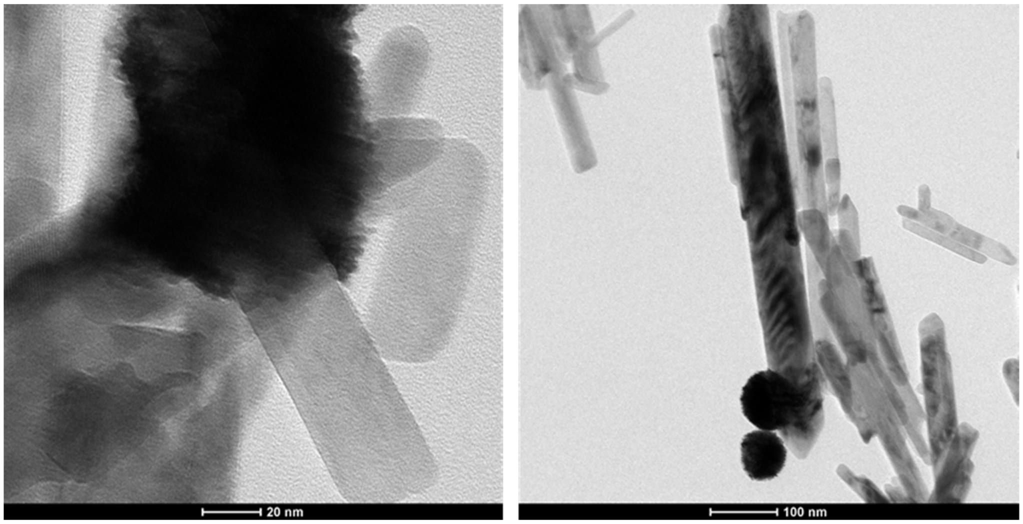

FigS1: TEM pictures of the aggregated platinum nanoparticles on the surface of the CrO_2 nanowires.

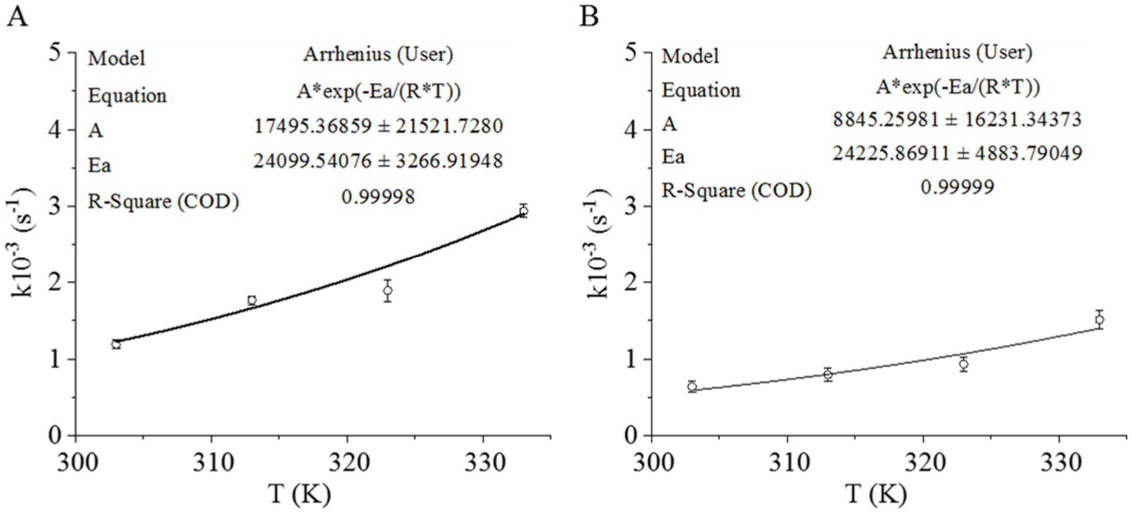

FigS2: Calculation of the activation energies (Ea) by non-linear regression of the Arrhenius plots in the case of the Pt/CrO_2 (A), Pd/CrO_2 (B) catalysts.

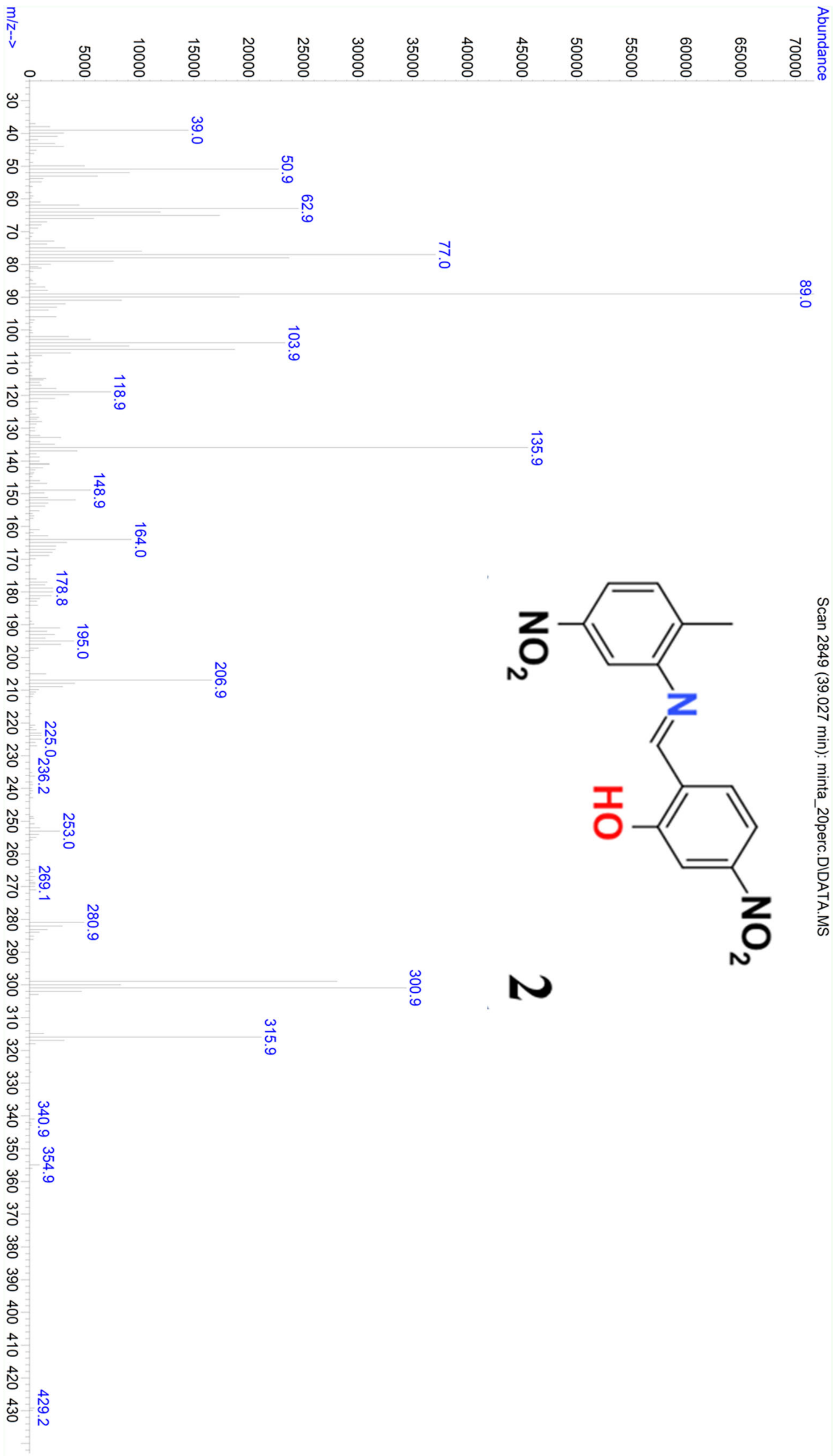

FigS3: Mass spectrum of the 2-[(*E*)-[(2-methyl-5-nitrophenyl)imino]methyl]-5-nitrophenol

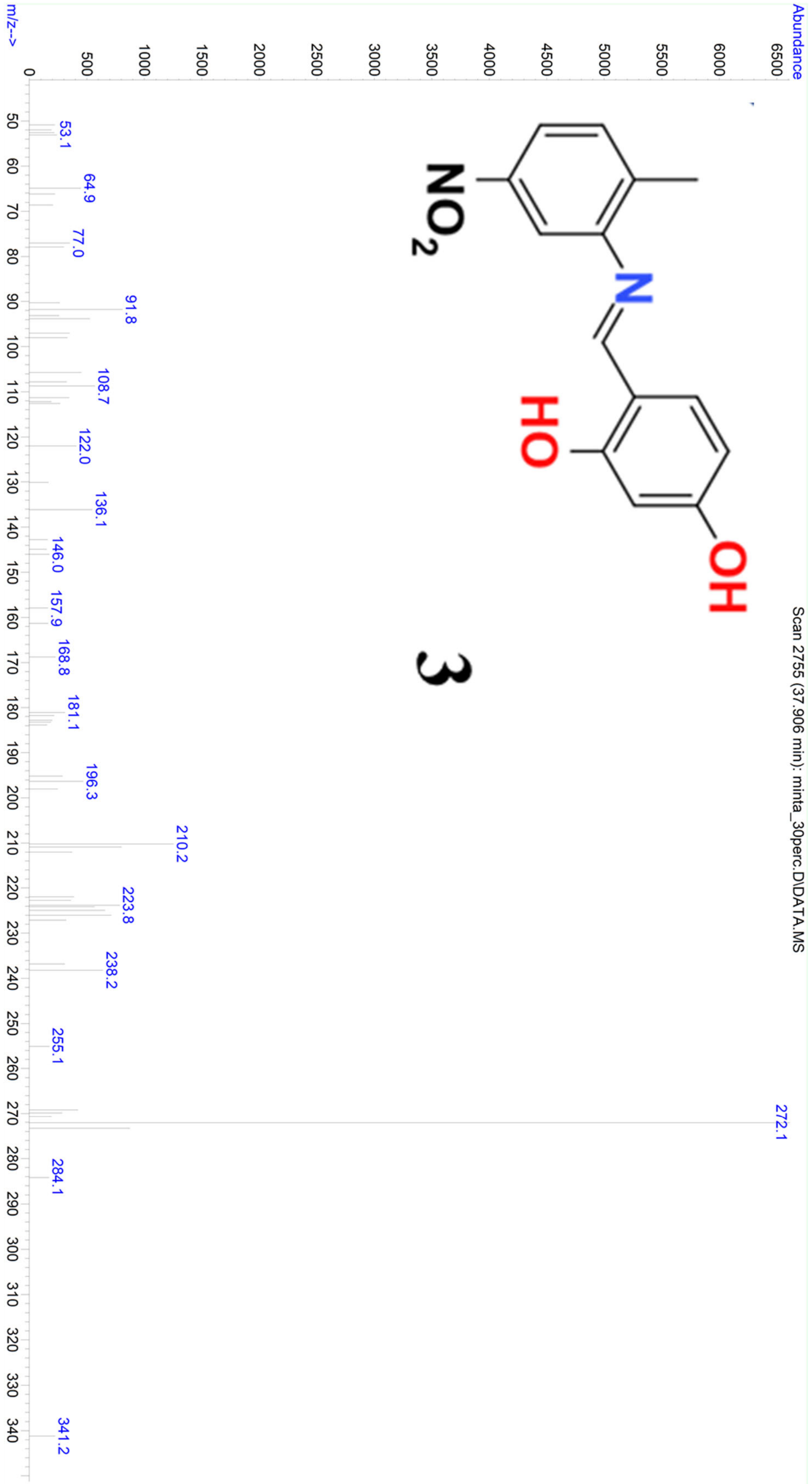

FigS4: Mass spectrum of the 4-[(*E*)-[(2-methyl-5-nitrophenyl)imino]methyl]benzene-1,3-diol

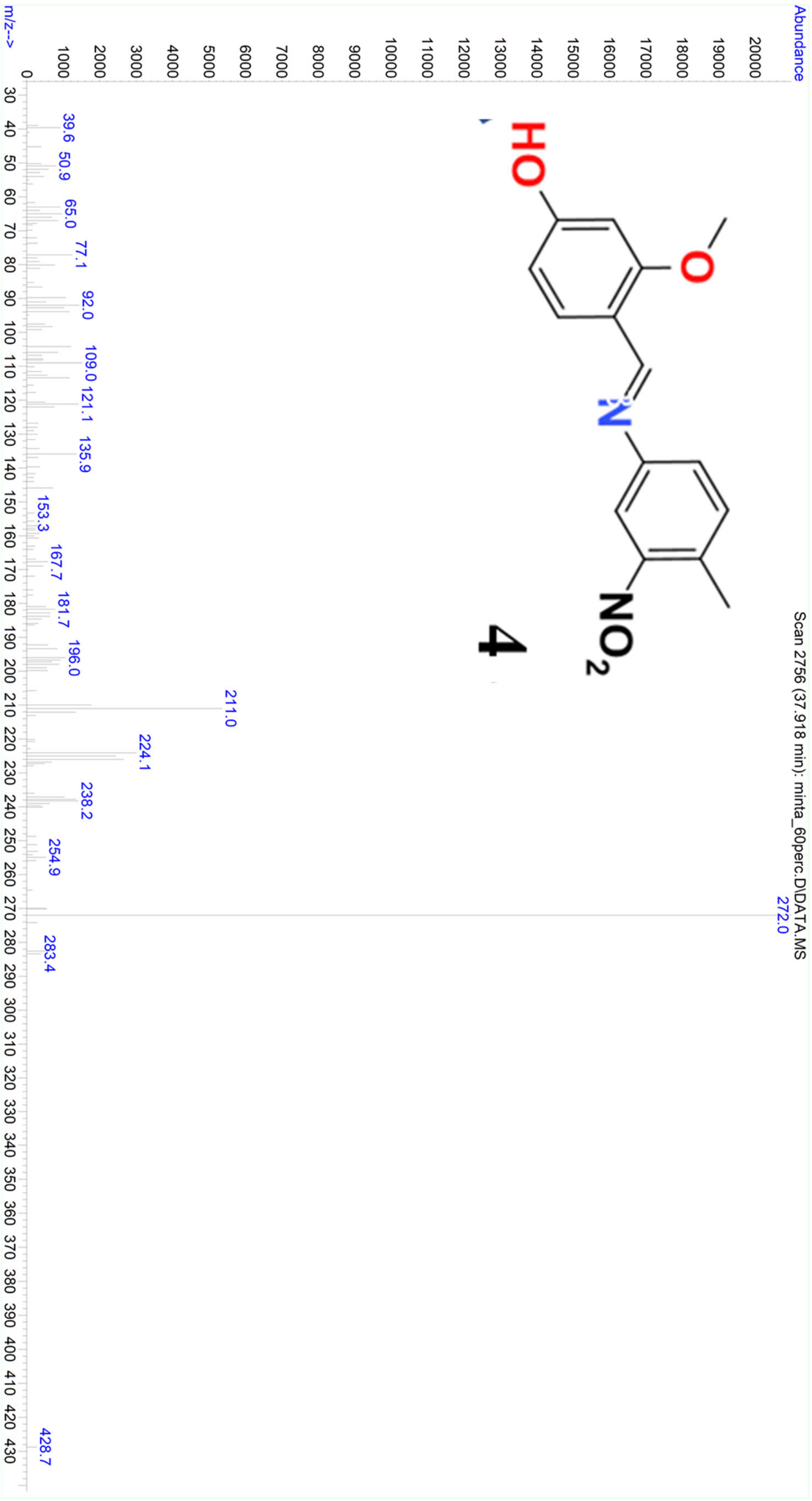

FigS5: Mass spectrum of the 3-methoxy-4-[(*E*)-[(4-methyl-3 nitrophenyl)imino]methyl]phenol
